# Short Chain Fatty Acids (SCFAs) Are the Potential Immunomodulatory Metabolites in Controlling *Staphylococcus aureus*-Mediated Mastitis

**DOI:** 10.3390/nu14183687

**Published:** 2022-09-06

**Authors:** Muhammad Akhtar, Syed Umair-Ali-Shah Naqvi, Qiyao Liu, Hong Pan, Ziyu Ma, Na Kong, Yan Chen, Deshi Shi, Muhammad Fakhar-e-Alam Kulyar, Jawaria Ali Khan, Huazhen Liu

**Affiliations:** 1 Key Laboratory of Agricultural Animal Genetics, Breeding and Reproduction of Ministry of Education, Huazhong Agricultural University, Wuhan 430070, China; 2 Hubei Hongshan Laboratory, Wuhan 430070, China; 3 The Cooperative Innovation Center for Sustainable Pig Production, Huazhong Agricultural University, Wuhan 430070, China; 4 Department of Zoology, Government Islamia Graduate College, Civil Lines, Lahore 54000, Pakistan; 5 Department of Preventive Veterinary Medicine, College of Animal Science and Veterinary Medicine, Huazhong Agricultural University, Wuhan 430070, China; 6 Department of Clinical Veterinary Medicine, College of Animal Science and Veterinary Medicine, Huazhong Agricultural University, Wuhan 430070, China; 7 Department of Veterinary Medicine, Faculty of Veterinary Science, University of Veterinary and Animal Sciences, Lahore 54000, Pakistan

**Keywords:** short chain fatty acids, *Staphylococcus aureus*, mastitis, immunomodulation, immune response

## Abstract

Mastitis is an emerging health concern in animals. An increased incidence of mastitis in dairy cows has been reported in the last few years across the world. It is estimated that up to 20% of cows are suffering from mastitis, causing incompetency in the mucosal immunity and resulting in excessive global economic losses in the dairy industry. *Staphylococcus aureus (S. aureus)* has been reported as the most common bacterial pathogen of mastitis at clinical and sub-clinical levels. Antibiotics, including penicillin, macrolides, lincomycin, cephalosporins, tetracyclines, chloramphenicol, and methicillin, were used to cure *S. aureus*-induced mastitis. However, *S. aureus* is resistant to most antibiotics, and methicillin-resistant *S. aureus* (MRSA) especially has emerged as a critical health concern. MRSA impairs immune homeostasis leaving the host more susceptible to other infections. Thus, exploring an alternative to antibiotics has become an immediate requirement of the current decade. Short chain fatty acids (SCFAs) are the potent bioactive metabolites produced by host gut microbiota through fermentation and play a crucial role in host/pathogen interaction and could be applied as a potential therapeutic agent against mastitis. The purpose of this review is to summarize the potential mechanism by which SCFAs alleviate mastitis, providing the theoretical reference for the usage of SCFAs in preventing or curing mastitis.

## 1. Introduction

Mastitis is considered the most common and predominant infection in dairy cattle around the globe. It is characterized by inflammation of mammary glands, impaired milk quality and production, and compromised animal wellbeing. It is estimated that up to 20% of cows are suffering from mastitis [1]. Besides, it causes incompetency in mucosal immunity and is responsible for substantial economic losses in dairy cows [2,3,4]. A wide range of intruded pathogens could be responsible for mastitis etiology. However, *Staphylococcus aureus (S. aureus)* is the most common infectious and opportunistic pathogen in dairy cows [2]. It contributes to mastitis initiation and leads to compromised host immunity [5]. Immunocompromised animals are more susceptible to mastitis and other infections [6]. Antibiotics, such as penicillin, macrolides, lincomycin, cephalosporins, tetracyclines, chloramphenicol, and methicillin, are usually applied against *S. aureus* infection [7]. However, methicillin-resistant *S. aureus* (MRSA) is causing a substantial failure of preventive strategies and could lead to a higher mortality rate in humans due to dairy milk consumption, which contains antibiotic resides [8,9,10]. Therefore, for preventing and curing mastitis, searching for an alternative to antibiotics has become an emerging concern.

Short-chain fatty acids (SCFAs) are produced by the gut microbiota through fermentation and play a crucial role in host/pathogen interaction [11,12]. Formic acid, acetic acid, propionic acid, butyric acid, valeric acid, and caproic acid are the important SCFAs consisting of carbon (C)1, C2, C3, C4, C5, and C6, respectively [13,14]. It has been reported that SCFAs could regulate energy metabolism, train immunity, suppress inflammatory reactions, stabilize gut eubiosis, and maintain gut integrity [15,16]. Recent studies emphasized that SCFAs decreased *S. aureus* internalization in the mammary glands and inhibited *S. aureus*-induced mastitis [12,17], suggesting the potential role of SCFAs as immunomodulatory metabolites against *S. aureus* infection. This review summarizes the potential mechanism of SCFAs in alleviating mastitis by encountering *S. aureus* pathogenesis, modulating gut-mammary dysbiosis, maintaining the blood–milk barrier, and regulating host innate/adaptive immune responses.

## 2. SCFAs Prevent Mastitis by Encountering *S. aureus* Pathogenesis via Challenging Its Adhesion, Invasion, and Evasion

Among all *Staphylococcus* species, *S. aureus* is causing a comparatively more infection burden than other species because of persistent host tissue colonization [18]. A recent study documented a variety of *S. aureus* virulence factors that influence its attachment, penetration, and evasion from the host immune system and help to survive in the microenvironment of mammary glands [19,20]. In fact, *S. aureus* bears many virulence cell wall anchored (CWA) proteins, i.e., near iron transporter (NEAT), G5-E repeat, and microbial surface component recognizing adhesive matrix molecule (MSCRAMM) [21]. Transcriptional sigma factor B (SigB) promotes the adhesion for colonization, and accessory gene regulator (Agr) helps in invasion [22]. Clumping factor B (ClfB) significantly helps bacterial adhesion, thus contributing to *S. aureus* pathogenicity [23,24]. Likewise, serine-aspartate repeat-containing protein C (SdrC) is critical to induce the formation of biofilm on *S. aureus* surface [25]. Moreover, capsular polysaccharide (CP) and protein A are also vital immune-evasion antigens of *S. aureus*. Of these two antigens, *S. aureus* crucially expresses protein A to increase pathogenicity via suppressing the host immune system [26]. Therefore, countering *S. aureus* intrusion into the intra-mammary microenvironment could be a potential strategy for mastitis prevention [27].

**Figure 1:**
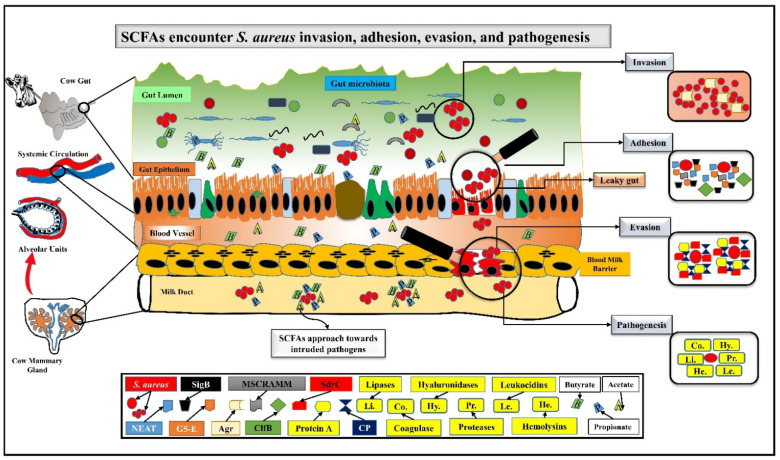
SCFAs encounter *S. aureus* invasion, adhesion, evasion, and pathogenesis.

Acetate, butyrate, and propionate are the important SCFAs present both in the host gut and milk, have the ability to diffuse across the cell membrane of bacteria, and exert active immunomodulatory and anti-inflammatory effects [28,29]. It is believed that SCFAs get entry into bacterial cells as an undissociated state, and its dissociation after entry causes intracellular acidification, predicting to inactivate the *S. aureus* virulence and protect the host from mastitis [30]. At 5 mM concentrations, sodium acetate inhibits 80% of *S. aureus* invasion and internalization in bovine mammary epithelial cells (bMECs) [28]. Further, *Lactococcus* with the ability of SCFAs production could inhibit *S. aureus* invasion in bMECs during mastitis [31]. The inhibitory mechanism for the adhesion, invasion, and evasion by SCFAs is interesting. For instance, SCFAs remarkably decreased the expression of the adhesion molecules, i.e., intercellular adhesion molecule 1 (ICAM1) [32] and vascular cell adhesion molecule 1 (VCAM1) [33], preventing *S. aureus* attachment. Sodium phenylbutyrate significantly stimulated bovine mammary alveolar (MAC-T) cells to increase host defense peptide (HDP) to encounter the invasion of *S. aureus* inflammation [34]. Another report indicated that sodium butyrate could inhibit ~50% of *S. aureus* invasion in bMECs, demonstrating the immunomodulating potential of butyrate [35]. Moreover, another study reported the remarkable inhibitory and immunomodulatory effects of sodium propionate (27–55%) and sodium hexanoate (39–65%) on *S. aureus* internalization in bMECs [12]. Besides, sodium butyrate significantly downregulated the mRNA expression of *S. aureus* invasion and biofilm formation related genes ClfB and SdrC, thus encountering *S. aureus* internalization in bMECs [36]. Furthermore, sodium acetate [28] and sodium propionate or hexanoate [12] significantly modulated the gene expression of tracheal antimicrobial peptide (TAP) or bovine neutrophil beta-defensin 5 (BNBD5) in bMECs and inhibited *S. aureus* internalization. Similarly, *S. aureus* invasion could be challenged by the sodium butyrate-mediated increased production of NO in bMECs, which has been found to be noxious for bacteria [35], and sodium butyrate via TLR2/p38 also activated bMECs to promote antimicrobial defense [37]. Although these studies indicated that SCFAs could modulate *S. aureus* adhesion, invasion, and evasion and could be applied to prevent *S. aureus*-mediated mastitis (Figure 1), ascertaining the exact mechanism for SCFAs inhibiting *S. aureus* pathogenesis is still warranted.

*S. aureus* is an opportunistic pathogen and can colonize in the host tissues persistently. *S. aureus* has particular virulence factors for invasion, adhesion, and evasion, i.e., SigB, Agr, ClfB, and SdrC for biofilm, NEAT, G5-E repeat, and MSCRAMM for anchoring, and CP and protein A for pathogenesis. *S. aureus* also secrets coagulase, hyaluronidases, lipases, proteases, leukocidins, and hemolysins, which destroy the mammary tissues and degrade the epithelium. As the gut becomes leaky and pathogens rush intruding to the systemic circulation, SCFAs start struggling to challenge the invaders. Acetate, butyrate, and propionate are directed towards pathogens through blood as well as via the milk duct. SCFAs also modulate TAP, BNBD5, and NO expression and stop biofilm formation via downregulating ClfB and SdrC genes expression. Probably, they themselves try to stop pathogenic dissemination, and provide a suitable environment for leukocytes to kill/engulf these pathogens.

## 3. SCFAs Ameliorate Mastitis by Modulating Gut-Mammary Dysbiosis

Recently, the gut microbiota has seen increasing attention worldwide as it can maintain a homeostatic balance between health and disease [38]. Any disruption in the homeostasis of gut microbiota and host immune system is called “gut dysbiosis”, which is significantly associated with mastitis in animals [39,40]. In gut dysbiosis, the pathogen’s abundance was found more than the commensal abundance [40], which disrupted the gut epithelial barrier, and live bacteria could be transferred to mammary glands via peripheral or lymphatic circulation [41]. Pathogens or their toxic metabolites, i.e., lipopolysaccharide (LPS) intrude into blood circulation by crossing the disrupted gut epithelial barrier, travel to mammary glands, start colonization, cause mammary dysbiosis and tissue injury, and destabilize the immune response, which eventually leads to mastitis [41,42,43,44]. Rumen health is also critical in mastitis. A recent study revealed that the high concentrate diet-mediated subacute rumen acidosis (SARA) induced mastitis in Holstein Frisian cows. SARA increased the permeability of rumen, gut, and blood milk barriers, facilitating the translocation of LPS from the rumen to the mammary glands, which subsequently activated the systemic immune responses, exacerbating the tissue injuries of mammary glands, indicating how endogenous and exogenous pathogenic factors cumulatively contribute to mastitis through gut–mammary axis [42]. Strangely, a low pH and higher concentrations of SCFAs and LPS are found in high concentrate diet-induced SARA, and monocarboxylate transporter 1 (MCT1) that is essential to bind with SCFAs is also reduced, which further decreases the SCFAs absorption [45]. However, maintaining the moderate concentrations of SCFAs in the rumen could promote the epimural microbiota and regulate gut immune homeostasis [46]. Other studies also described that mammary dysbiosis could result in increased opportunistic and contagious pathogens, i.e., *Mycoplasma* spp., *Corynebacterium bovis*, *Streptococcus dysgalactiae*, *Streptococcus agalactiae,* and *Staphylococcus aureus* in the cow udder during mastitis [47]. Pang and co-workers found high a prevalence of *Rikenella*, *Bacteroides*, *Ruminococcus*, *Alistipes,* and *Prevotella* in the milk samples, which are typically gut-associated genera and might be reached in mammary glands via an entero-mammary pathway [44]. Likewise, a recent study reported that the prevalence of *S. aureus* ranges from 2% to 50% in clinical mastitis [48]. On the other hand, it is also found that SCFAs producing gut commensals (*Clostridium tyrobutyricum*) could also contribute to reducing blood-milk barrier permeability, mammary gland damages, and pro-inflammatory cytokine secretion, thus inhibiting *S. aureus*-induced mastitis [17]. These reports indicate that gut pathogens and their metabolites (LPS) remarkably contribute to the development of mastitis, while gut commensals (beneficial bacteria) and their metabolites (SCFAs) predominantly prevent mastitis. In fact, understanding the gut mammary axis during mastitis is critical, and therefore, controlling gut-mammary dysbiosis could ultimately prevent mastitis occurrence.

**Figure 2:**
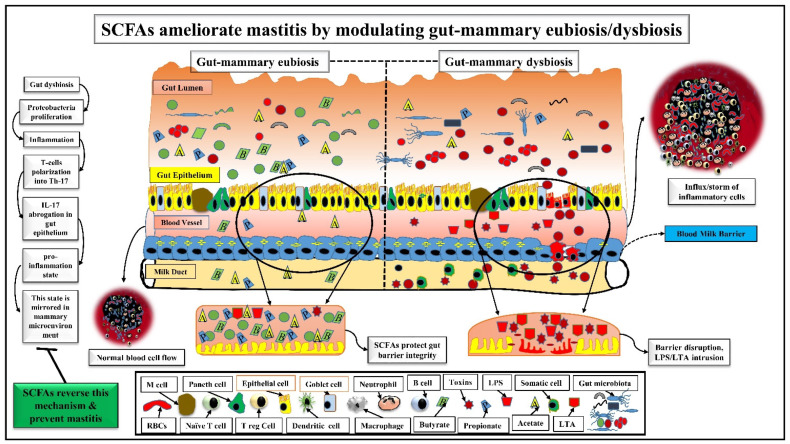
SCFAs ameliorate mastitis by modulating gut-mammary eubiosis/dysbiosis.

Recent studies described that SCFAs, along with their producers (gut commensals), could reduce gut dysbiosis and maintain gut homeostasis [49]. Bacteroidetes and Firmicutes are the major SCFAs producers (*Akkermansia*, *Ruminococcus*, *Eubacterium*, *Clostridium*, *Prevotella*, and *Veillonella*, etc.) that principally maintain gut eubiosis and produce butyrate and propionate that prevent udder from mastitis [17,50]. *Lactobacilli* are effective probiotics that enhance butyrate production via cross-feeding the gut commensals and decrease gut dysbiosis [51]. Therefore, *Lactobacilli* are useful in treating *staphylococcal* mastitis [52]. It is found that the intramammary administration of *Lactobacilli* might be effective in ruminants, but further research is still warranted. Thus, typically inhabiting commensal microbiota in mammary glands defends against *S. aureus* mastitis by preventing mammary-dysbiosis [52,53]. Another study demonstrated that SCFAs producers could prevent gut dysbiosis and are negatively associated with somatic cell counts (SCC) in milk during mastitis [54]. Butyrate ameliorates gut dysbiosis by increasing beneficial bacteria that produce SCFAs and decreasing harmful bacteria that promote inflammation [55]. Moreover, dysbiosis-mediated inflammation allows pathogenic microbiota (Proteobacteria) to facilitate T-cells polarization into Th-17 cells following IL-17 abrogation in the gut epithelium, which further increases bacterial colonization and pro-inflammation state in the gut. Th-17-derived pro-inflammation state in the gut is mirrored in mammary glands, which facilitates lymphocyte infiltration that starts producing IL-17 through extracellular signal-regulated kinase (ERK) signaling [56]. As SCFAs have the potential to modulate Th-1, Th-2, and Th-17 cells activation and polarization [57], thus, it is predicted that SCFAs and their producers could regulate the above mechanism reversely, maintain gut-mammary eubiosis, and prevent mastitis by regulating immune homeostasis. All these findings suggest the dynamic protective effects of SCFAs and their producers in the gut–mammary dysbiosis. These studies also describe that an appropriate gut-mammary physiology promotes intestinal homeostasis/eubiosis and inhibits inflammation/dysbiosis in mammary glands through immunomodulatory pathway (Figure 2).

Gut-mammary dysbiosis and inflammation are represented on the right side of the figure. Gut dysbiosis increases pathogenic invaders and decreases the commensals in the gut lumen. As a result, gut pathogens start their proliferation and initiate a low-grade inflammation, which damages the gut epithelial barrier, and several pathogens including *S aureus* (shown in red-color-filled circles) also intrude into the systemic circulation. After their intrusion, a storm of inflammatory cells initiates at the inflammatory injury site. Bacteria-released LPS, LTA, and toxins in the host circulation damage the blood milk barrier and get entry into the milk duct, where somatic cells also rush towards the inflammation site. On the other hand, gut-mammary eubiosis and homeostasis are represented on the left side. Here, the gut lumen is enriched with beneficial bacteria (shown in the green color) that maintain homeostasis. As a result, the gut epithelial barrier remains intact. Although some pathogenic bacteria, i.e., *S aureus* still exist in the gut lumen, SCFAs and their producers prevent their invasion towards gut epithelial brush. Some commensals and SCFAs could also translocate into the milk duct and maintain homeostasis in the mammary microenvironment. A sequential representation of how SCFAs could ameliorate mastitis by modulating gut–mammary dysbiosis is also given on the extreme left-side of the figure.

## 4. SCFAs Distinctly Participate in the Maintenance of Blood Milk Barrier and Mammary Gland Structure

The blood–milk barrier is a structural part of mammary glands that restricts the microbial invasion and inhibits luminal milk components leakage via epithelial tight junctions (T.Js.). It works well during normal physiological conditions and protects milk quality and host health. However, the integrity of these junctions is compromised during the infection, which leads to multiple complications, including the intrusion of infectious entities that initiate inflammation [58]. Years ago, it was reported that the blood milk barrier in mammary glands became leaky during mastitis in pregnant animals [59], and the disrupted barrier facilitates pathogen’s proliferation in mammary gland tissues, which promotes inflammation. For instance, *S. aureus* secretes coagulase, hyaluronidases, lipases, proteases, leukocidins, and hemolysins, which destroy the mammary tissues and degrade the epithelium of alveoli cisterns and ducts and disseminates inflammation [60,61]. As reported that gut microbiota plays an essential role in maintaining the blood-testis barrier (B.T.B.) and blood-brain barrier (B.B.B.). Likewise, a recent study described that gut microbiota alteration is directly linked with the permeability of the blood-milk barrier. It also alters the neutrophils threshold, whose influx via crossing the blood-milk barrier is increased, thus aggravating inflammation, i.e., mastitis [62,63]. Therefore, maintaining blood–milk barrier integrity and regulating its permeability would prominently promote the mammary gland’s functionality.

Recent evidence suggests that SCFAs are the most common gut microbiota metabolites, which are an essential source of energy for the proteins of blood-milk barrier [64]. For instance, *Clostridium tyrobutyricum* is a typical butyrate-producing gut bacterium, which has a protective role against *S. aureus* mastitis via reducing the permeability of the blood–milk barrier and strengthening T.Js. proteins [17]. Zona occludens-1 (ZO-1), claudin-3, and occludin are the structural proteins of T.Js. between mammary epithelial cells and any damage in these proteins could directly disturb the functions of T.Js. [65,66]. SCFAs could markedly facilitate T.Js. assembly and improve barrier functions. For instance, butyrate is a good energy source for T.Js. [67], and sodium propionate treatment enhanced gene expression of claudin-3 and occludin in bMECs, possibly to maintain blood-milk barrier integrity against mastitis [68]. Furthermore, lipopolysaccharide (LPS) is a typical toxin of Gram-negative bacteria, which significantly decreases claudin-3, and disturbs occludin localization, thus damaging T.Js. structure in mammary glands, and causes mastitis [69]. It is demonstrated that propionate inhibited LPS-induced mastitis via regulating the permeability of the blood milk barrier [70]. It is also reported that LPS triggers fluorescein iso-thiocyanate conjugate (FITC)-albumin to migrate from interstitium to the alveolar lumen, which aggravates disruption of T.Js. However, the treatment with sodium butyrate reduced the LPS-induced FITC-albumin leakage, and protected the normal structure of T.Js., thus preventing mastitis [71]. Furthermore, an in vitro/in vivo study demonstrated that butyrate induced innate lymphoid cells and CD4^+^ T cells to produce IL-22 via GPR41 and also inhibited histone deacetylase (HDAC). In this study, it is described that signal transducer and activator of transcription 3 (STAT3) and mechanistic target of rapamycin (mTOR) regulated aryl hydrocarbon receptor (AhR) expression and hypoxia-inducible factor 1α (HIF1α) transcription. Thus, butyrate produced IL-22 via STAT3 and mTOR regulation. These findings suggested that butyrate promoted intestinal homeostasis and barrier functions through increasing HIF1α binding with IL-22 promoter. The protein/mRNA expression of IL-22 exhibited that butyrate, acetate, and propionate induced IL-22 production [72]. Thus, an increased IL-22 level in milk helps to recover mammary gland structure by improving barrier function and by eliminating the bacteria from the mammary gland microenvironment [73]. Cumulatively, these reports suggest that maintaining the blood milk barrier is critical for the normal physiology of mammary glands, and SCFAs could help improve the integrity of the blood milk barrier to inhibit the internalization of infectious entities.

Additionally, it has also been recognized that intestinal epithelial cells (goblet cells, M cells, absorptive enterocytes, enteroendocrine cells, and Paneth cells) firstly confront the invading bacteria and their released toxins and SCFAs are important players to recover the functions of intestinal epithelial cells [74,75]. Recent findings demonstrated that butyrate is absorbed through the transporters, i.e., monocarboxylate transporter 1 (MCT1) and sodium coupled monocarboxylate transporter 1 (SMCT1), signaled through G-protein coupled receptors (GPRs)/free fatty acid receptors (FFARs), i.e., GPR41/FFAR3, GPR43/FFAR2, and GPR109A, and controlled the immune functions of these cells by inducing the genes that encode the proteins of T.Js. [49]. For instance, butyrate and acetate increased the differentiation of goblet cells and promoted mucus production, creating a protective blanket on the mucosa and maintaining barrier integrity [74]. Another study described that butyrate and propionate augmented the differentiation of goblet, enterocytes, enteroendocrine, Paneth, and M cells by increasing the production of β-defensin [76,77], promoting the potential of intestinal epithelial cells against intruded pathogenic bacteria. Thus, SCFAs impart protective effects on the epithelial cells and help to maintain barrier integrity.

## 5. SCFAs Distinctly Alleviate Mastitis by Regulating Immune Homeostasis

**Figure 3:**
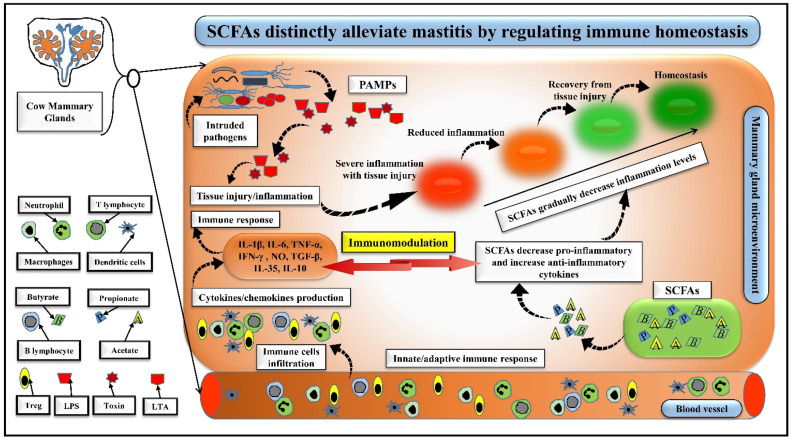
SCFAs distinctly alleviate mastitis by regulating immune homeostasis.

Stabilizing immune homeostasis during pathogenic inflammation is crucial for the host. During mastitis, immune homeostasis is regulated by innate and adaptive immune responses, which respond quickly and synergistically to the invading microbes and/or bacterial antigens [78,79]. Evidence suggests that SCFAs could alter inflammation levels by modulating the innate and adaptive immune responses [57], facilitating the development of a therapeutic strategy to control mastitis in animals [35]. It has been reported that SCFAs could regulate the immune homeostasis and prevent tissue injury through several ways. Firstly, SCFAs could effectively modulate the innate immune response in the mammary glands by reducing cell adhesion and chemotaxis, and regulating the physiology of monocytes, neutrophils, macrophages, and lymphocytes, which secrete some inflammatory markers, i.e., cytokines and/or chemokines [12,80]. SCFAs excellently decrease the extent of these inflammatory markers whose secretion correlates to the circulating levels of SCFAs [57]. Accumulated evidence suggests that SCFAs inhibit inflammation through binding their G-protein coupled receptors (GPRs)/free fatty acid receptors (FFARs), i.e., GPR41/FFAR3, GPR43/FFAR2, and GPR109A on the immune cells [17,50]. For instance, Sodium propionate decreased the activity of an inflammatory marker, myeloperoxidase (MPO) by regulating neutrophil influx [70]. Further, butyrate decreased IL-6, COX-2, & IL-1β secreted by bovine macrophages [81]. An in-vitro study described how sodium propionate/sodium hexanoate with the dose of 0.25–1 mM inhibited *S. aureus* internalization in bMECs via activating their GPR41 and/or GPR43 [12]. Thus, SCFAs uniquely modulate innate immunity during inflammation and maintain immune homeostasis in the mammary glands. Secondly, SCFAs importantly regulate the T lymphocyte’s functions in the adaptive immune system [57] and enhance T lymphocyte differentiation into regulatory T cells (Treg) or effector T lymphocytes [82] by binding through their GPRs with these cells [57]. Importantly, acetate regulates T lymphocyte proliferation via HDAC and mTOR and upregulates IL-10 production [82]. Furthermore, butyrate decreases chemokine receptor-2 (CXCR-2) expression in lymphocytes and regulates T cell recruitment to the inflammation site [83]. B-lymphocytes play an essential role in IgA production against invading pathogenic bacteria [84], and SCFAs promote B cell activity and differentiation [85]. Sodium butyrate exerts better effects on immune modulation via decreasing the leukocyte infiltration and somatic cell counts in milk [86]. These reports indicate that an effective immune response in the host immune system is essential to combat the intruding pathogens in the mammary gland microenvironment, and SCFAs-mediated immunomodulation plays a substantial role in regulating the immune homeostasis and preventing tissue injury (Figure 3).

Intruding pathogens either from blood circulation due to gut–mammary dysbiosis or from teat/mammary gland orifice released PAMPs (LTA, LPS or toxins) in the mammary gland’s microenvironment. As a result, immune cells promptly respond to the secreted PAMPs, and macrophages, neutrophils, Treg, and T or B lymphocytes induce an overwhelming storm of inflammatory mediators, including pro- and anti-inflammatory cytokines as an innate and adaptive immune response, which leads to inflammation and tissue injury. SCFAs absorbed from the entero-mammary pathway or produced from mammary microbiota rush towards the inflammatory site to challenge the inflammatory injury. SCFAs regulate the influx of immune cells to the inflammation site, restricting those involved in a pro-inflammation state and promoting those involved in an anti-inflammation state, thus effectively and gradually decreasing inflammation levels and tissue injury to the homeostasis level as represented in the figure with red, light red, light green, and green colors (circles). Finally, SCFAs play a potential immunomodulatory role in regulating immune homeostasis.

**Figure 4:**
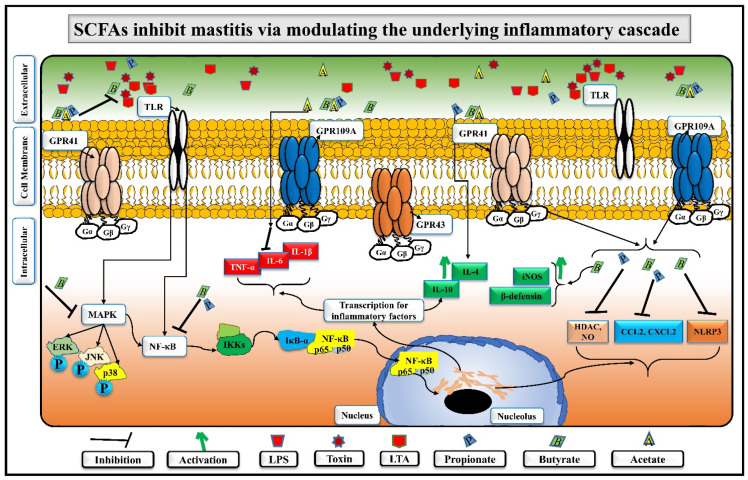
The extracellular and intracellular proposed mechanism of SCFAs inhibiting MAPKs/NF-κB inflammatory cascade.

Thirdly, it has been reported that mitogen-activated protein kinases (MAPKs) and nuclear factor kappa B (NF-κB) are the central inflammatory signalling pathways, which could be targeted in mastitis [5]. Pathogen-associated-molecular-patterns (PAMPs), i.e., LPS-, lipoteichoic acid (LTA)-, or other toxin-stimulated secretion of inflammatory cytokines are often observed through MAPKs/NF-κB/HDAC pathways via pattern recognition receptors (PRRs), i.e., toll-like receptors (TLRs) in bovine mastitis [5,70]. It is observed that pathogenic activated NF-κB and MAPKs pathways caused overexpression of pro-inflammatory cytokines (IL-1β, IL-6, and TNF-α) in mastitis [87]. However, SCFAs could inactivate NF-κB in mastitis and regulate host immunity and homeostasis [88]. For instance, sodium butyrate at 50, 100, and 200 mg/kg^−1^ suppressed IL-1β, IL-6, and TNF-α by inhibiting NF-κB and MAPKs pathways [11,71]. Propionate also reduced mastitis in bMECs by inhibiting important chemokines, i.e., C-C motif ligand 2 (CCL2) and CXC motif chemokine ligand 2 (CXCL2) [68]. Recent evidence suggests that SCFAs also regulate the inhibition of histone deacetylase (HDAC), which affects the expression of certain genes responsible for regulating immune homeostasis and is linked with nitric oxide (NO) and NF-κB signalling [89,90]. Propionate decreased the inflammatory factors (IL-1β, IL-6, and TNF-α) via inhibiting NF-κB and HDAC in in-vitro and in-vivo mastitis models [70]. Likewise, butyrate and propionate inhibited HDAC and NO production and reduced *S. aureus*-lipoprotein-induced inflammation in RAW 264.7 cells [28,91]. Inhibition of NF-κB/NLRP3 inflammatory pathways with sodium phenyl butyrate in LTA-induced bovine mammary alveolar cells has also been reported [34], emphasizing the anti-inflammatory role of sodium phenyl butyrate in mastitis. The attenuation of this pathway was also demonstrated by other scientists in bMECs, who found that sodium propionate [68] and sodium butyrate [88] significantly inhibited the activity of HDAC, the activation of NF-κB, and the secretion of TNF-α, IL-6, and IL-Iβ, suggesting an anti-inflammatory potential of SCFAs. On the other hand, propionate and butyrate promote IL-10 production in immune cells, which is an essential anti-inflammatory cytokine [92]. Interestingly, an increased level of IL-10 could support regulating immune homeostasis and lead to a better immunomodulatory effect during inflammation [50]. Another experiment in bMECs revealed that butyrate significantly inhibited *S. aureus* by enhancing β-defensin and inducible nitric oxide synthase (iNOS) expression [35]. The above-cited literature suggests that SCFAs could inhibit inflammation through modulating underlying inflammatory and immunomodulatory signalling pathways (Figure 4). Interestingly, it is established that in LPS or LTA-induced inflammation, MAPKs and NF-κB inflammatory pathways are often activated through TLRs expression [93]. As PAMPs initiate TLRs for inflammation dissemination in the tissues, the above discussion indicates that mastitis inhibition with SCFAs would definitely suppress the TLRs expression indirectly in the mammary glands. However, investigation of the exact mechanism of how SCFAs inhibit/suppress/correlate with TLRs to minimize inflammation is still warranted.

In extracellular matrix, the gut pathogens-produced LPS, LTA, and other toxins are always present and ready for entry into susceptible cells, while gut commensals-produced useful metabolites exist in the same matrix and are also continuously protecting the cells from the harmful effects of PAMPs by surrounding the plasma membrane. After availing the suitable opportunity in immunocompromised/immunosuppressed host, LPS, LTA, and other toxins bind with their related PRRs, i.e., TLRs and trigger MAPKs/NF-κB/NLRP3/HDAC pathways, which stimulate the transcription of several inflammatory genes in the nucleus. As a result, an overwhelming storm of pro-inflammatory cytokines exacerbates the inflammatory state. On the other hand, SCFAs, i.e., acetate, propionate, and butyrate, bind with their related GPRs, i.e., GPR41, GPR43, and GPR109A, inactivate/suppress the pathogenic virulence PAMPs through enhancing iNOS and β-defensin secretion, and support the secretion of some anti-inflammatory cytokines, thus helping to reduce the pro-inflammation state by regulating the immune response and inhibiting *S. aureus* internalization.

## 6. Conclusions and Future Perspectives

Mastitis is the inflammation of mammary glands and is recognized with diverse histopathological findings in dairy cows. It is characterized by huge economic losses in the dairy industry in terms of decreased milk yield/quality and treatment and/or death costs. Many pathogenic bacteria could cause mammary gland inflammation, but *S. aureus* is recognized as the most common infectious pathogen causing mastitis in dairy cows. Although antibiotics have been applied for years in treating mastitis, their excessive usage has led the emergence of some resistant strains, challenging mastitis treatment. In this scenario, antibiotic resistant strains, especially MRSA, aggravate the reoccurrence, dissemination, and pathogenesis of mastitis. *S. aureus* impairs immune homeostasis and compromises host immunity, leaving the host more susceptible to other infections. Additionally, continuous consumption of antibiotics often kills beneficial gut commensals by increasing the gut pathogens, which severely disturb the ecology of the gut microenvironment, resulting in gut dysbiosis. Recent implications of gut microbiota-derived bioactive metabolites, SCFAs, in host immunomodulation could be facilitative in regulating host immunity and physiology. Accumulating evidence emphasizes the profound effects of SCFAs in treating *S. aureus*-induced mastitis.

This review summarizes that SCFAs and their producers maintain gut eubiosis in the gastrointestinal tract by facilitating the flourishing of beneficial (commensal) gut microbiota. On the one hand, SCFAs protect mucosal barrier integrity by supporting intestinal epithelial cells and inhibit *S. aureus* internalization to the mammary glands microenvironment by encountering its adhesion, invasion, and evasion. SCFAs also facilitate restoration of the blood–milk barrier functionality and reduce its permeability, thus inhibiting the intrusion of pathogens and their produced metabolites (LPS or LTA). On the other hand, SCFAs-mediated immunomodulation plays a substantial role in regulating the immune homeostasis appropriately and preventing tissue injury. Interestingly, SCFAs through their GPRs suppress *S. aureus*-mediated TLRs and inhibit the underlying inflammatory signalling cascade, including MAPKs, NF-κB, NLRP3, and HDAC. Moreover, SCFAs remarkably regulate innate and adaptive immune responses by suppressing the secretion of pro-inflammatory cytokines and promoting the release of anti-inflammatory cytokines. On the whole, these findings suggest that SCFAs have great immunomodulatory potential to maintain immune homeostasis and decrease the inflammatory tissue injuries in the mammary glands. Thus, SCFAs could be implicated in treating *S. aureus*-mediated mastitis. Therefore, focusing on immunomodulatory potential and considering SCFAs to replace antibiotics could be a considerable solution to the recent concern of antibiotic resistance.

## Data Availability

Not applicable.
